# Alfalfa Seed Decontamination in *Salmonella* Outbreak

**DOI:** 10.3201/eid0904.020519

**Published:** 2003-04

**Authors:** Christopher J. Gill, William E. Keene, Janet C. Mohle-Boetani, Jeff A. Farrar, Patti L. Waller, Christine G. Hahn, Paul R. Cieslak

**Affiliations:** * Oregon Department of Human Services, Portland, Oregon, USA;; † Tufts University-New England Medical Center, Boston, Massachusetts, USA; ‡ California Department of Health Services, Berkeley, California, USA; § California Department of Health Services, Sacramento, California, USA; ¶ Washington State Department of Health, Shoreline, Washington, USA; # Idaho Department of Health and Welfare, Boise, Idaho, USA

**Keywords:** Alfalfa, seed sprouts, salmonellosis, foodborne illness, salmonella outbreak, Oregon, seed disinfection, food safety

## Abstract

Based on in vitro data, the U.S. Food and Drug Administration recommends chemical disinfection of raw sprout seeds to reduce enteric pathogens contaminating the seed coats. However, little is known about the effectiveness of decontamination at preventing human disease. In 1999, an outbreak of *Salmonella enterica* serotype Mbandaka occurred in Oregon, Washington, Idaho, and California. Based on epidemiologic and pulsed-field gel electrophoresis evidence from 87 confirmed cases, the outbreak was linked to contaminated alfalfa seeds grown in California’s Imperial Valley. Trace-back and trace-forward investigations identified a single lot of seeds used by five sprout growers during the outbreak period. Cases of salmonellosis were linked with two sprout growers who had not employed chemical disinfection; no cases were linked to three sprout growers who used disinfection. This natural experiment provides empiric evidence that chemical disinfection can reduce the human risk for disease posed by contaminated seed sprouts.

Despite the popular perception of sprouts as health food, alfalfa and other varieties of raw seed sprouts are common vehicles for produce-associated bacterial foodborne illness. Although sprout-associated outbreaks have been reported since 1973 (*1*), back-to-back multinational outbreaks of gastroenteritis with *Salmonella enterica* serotypes Newport and Stanley in 1995 and 1996 (*2*,*3*) and the 1996 Sakai city outbreak of enterohemorrhagic *Escherichia coli* O157:H7 in >5,000 Japanese schoolchildren have refocused attention on the public health hazard posed by seed sprouts (*4*).

The risk for disease from sprouts is connected to seed production and distribution factors and the sprouting process itself. Seeds may be reared, harvested, milled, and sprouted locally or shipped globally to sprout growers; bacterial contamination may occur at any point in this chain (*5*). During germination, seeds are presoaked in water and then germinated in a warm, moist, aerated environment for 3 to 7 days. Replication of pathogens by three to five orders of magnitude may occur during sprouting, resulting in high pathogen levels on mature sprouts, despite the fact that initial densities are low and the pathogens dispersed irregularly throughout seeds (*6*). In an experimental model of seed contamination*, Salmonella* Stanley added to alfalfa seeds increased from 3.29✕10^6^ bacteria per gram of mature sprouts to 10^7^ bacteria per gram after 48–72 hours incubation, without affecting the appearance, smell, or taste of the sprouts (*6*).

Methods proposed to reduce the risk to human consumers include testing seeds, irrigation water, and sprouts for pathogens (*7*–*12*) and disinfecting seeds (*13*–*16*). In vitro, treating seeds with 20,000 ppm calcium hypochlorite [Ca(Ocl)_2_] pregermination reduces pathogen densities by up to 2.2 log (*13*,*14*). Higher concentrations of disinfectant or the use of concentrated acids, high temperatures, or bleaches reduces pathogen levels by >3 log; these treatments substantially reduce the proportion of seeds that germinate (*16*,*17*). Notably, none of these methods completely eliminates pathogens on seeds. Based on these data, the U.S. Food and Drug Administration (FDA) currently recommends that seeds be treated with 20,000 ppm Ca(Ocl)_2_ pregermination and that sprouts and spent irrigation water samples be periodically tested for enteric pathogens. The number of sprout growers (referred to as “sprouters”) who follow these guidelines, as well as the proportion of sprouts grown from disinfected seeds, is unknown.

*S*. Mbandaka, an uncommon serotype in Oregon, occurred from 1988 to 1998 at an average rate of 1.5 cases per year. From January to April 1999, the Oregon Department of Human Services (ODHS) conducted an outbreak investigation in response to a sharp increase in cases of *S*. Mbandaka in what soon proved to be a multistate outbreak. This investigation ultimately provided insight into the efficacy of seed disinfection.

## Methods

We defined case-patients as persons with culture-confirmed *S.* Mbandaka infection and onset of acute illness from January 1 to April 15, 1999. Cases were excluded if they were subsequently shown to have a pulsed field gel electrophoresis (PFGE) pattern that differed from the outbreak pattern. If the illness onset date was unknown, the specimen collection date minus 2 days was used instead.

To generate hypotheses about potential exposure vehicles, ODHS conducted open-ended interviews with the first 10 Oregon case-patients. Based on these interviews, we performed a 2:1 (controls:cases) age-matched case-control study using these cases. Age-matched controls were identified by sequentially adding or subtracting one digit from each case-patient’s telephone number prefix. Matched ranges were: 1–18, 19–50, and 51–100 years of age. Using a standardized food questionnaire, we asked respondents about consumption of food that typically carries *Salmonella* during the preceding week.

After alfalfa sprouts were implicated, we traced the source of sprouts from the case-patients to the brand of sprout’s point of production to identify a common source for the contaminated sprouts. Onsite investigations of an implicated sprouter were conducted in partnership with the Washington Department of Agriculture and the FDA. Seed, water, and sprout samples were collected for culture with sterile swabs and containers.

Because alfalfa seeds are often produced in bulk, we conducted trace-back and trace-forward investigations using purchase invoices and shipping records to identify other sprouters who may have purchased and used potentially contaminated seeds from the same production lot. Other sprouters who purchased this lot were asked about their use of the implicated lot, including quantities sprouted and their seed-disinfection practices. To identify additional cases, we queried health departments throughout the United States and Canada, with specific attention to states where seed from the implicated lot had been distributed.

### Laboratory Methods

All *Salmonella* isolates from patients were serotyped at the Oregon, California, Washington, and Idaho state laboratories. *Salmonella* cultures were performed according to standard methods (*18*,*19*). PFGE, by using the restriction enzyme *Xba*I (Boehringer Mannheim, Indianapolis, IN), was used to corroborate genetic relatedness of *S.* Mbandaka isolates according to the method of Tenover et al. (*20*). Given the rarity of *S*. Mbandaka, we did not consider it necessary to use two or more restriction enzymes to achieve a still higher degree of resolution with PFGE. Two *S.* Mbandaka isolates from 1997 to 1998 were used as reference standards.

### Statistical Methods

We calculated matched odds ratios, 95% confidence intervals, and p values using Exact (*21*). A two-tailed p value of <0.05 was considered significant. Using food frequency data from Oregon FoodNet showing that ~10% of Oregonians consume sprouts on a weekly basis (*22*), we performed a binomial calculation of the probability that the observed proportion of sprout exposure among cases was due to chance (*23*).

## Results

### Descriptive Epidemiology

**Figure 1:**
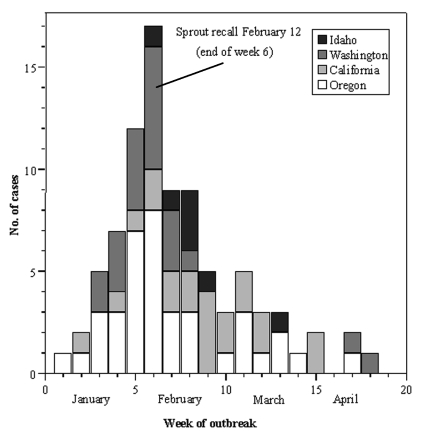
Epidemic curve of *Salmonella* Mbandaka outbreak, 1999. Line indicates the timing of the Oregon Health Division’s press release alerting the public of the outbreak. A lot L seed embargo and voluntary recall of brand X sprouts also occurred at this time.

From January 1 to April 15, 1999, a total of 89 cases of *S*. Mbandaka were identified in four states: Oregon (42 cases), Washington (19 cases), Idaho (7 cases), and California (21 cases) (Figure 1); 74% of the case-patients were women, and the median age was 28 years of age (range, 6 months to 98 years; interquartile range: 19–41 years). Two additional cases were not included because neither could be linked epidemiologically with the outbreak and both had different PFGE patterns. *S*. Mbandaka was cultured from 69 stool samples, 14 urine samples, three blood cultures, one abscess, one blood and stool sample, and one unspecified source. The association between female sex and urinary isolates tended towards significance (p=0.06, Fisher exact test). Two cases clustered within a household, but were not considered to represent secondary spread of the disease, as they occurred concurrently. No patients died.

Nine of the first 10 case-patients and none of 20 matched control-patients reported eating or handling alfalfa sprouts (matched odds ratios were undefined [zero in denominator]; 95% confidence interval 3.4 to ∞; p<0.001). No other food vehicles were statistically associated with illness. From the binomial theorem, given an expected frequency of 10%, the probability of 9 out of 10 persons reporting sprout consumption within the past week was <1/1000 (*22*).

### Identification of Sprout Source

**Figure 2:**
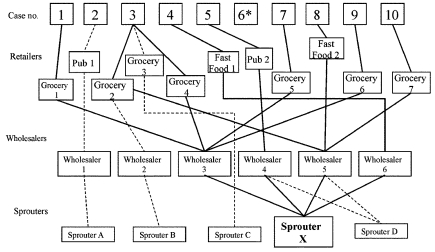
Trace-back investigations of Oregon cases of *Salmonella* Mbandaka. *Case-patient 6 recalled no sprout exposure. Solid lines indicate trace-back routes leading to sprouter X. Dashed lines indicate trace-back routes leading to other sprouters.

The first 10 Oregon case-patients reported eating sprouts from one or more of five growers (sprouters A, B, C, D, and X). Trace-back investigation showed that the common link between most cases was sprouter X (Figure 2). Eight of nine case-patients reported eating sprouts that could have come from sprouter X. Five of these eight cases had potential links to other sprouters in addition to sprouter X, while three of the eight cases could be traced only to sprouter X.

The index patient of *S.* Mbandaka provided a leftover sample of sprout X brand alfalfa sprouts. Cultures from this sample subsequently grew *S*. Mbandaka.

### Onsite Investigations at Sprouter X’s Facility

Based on these investigations, representatives of the Washington Department of Health and FDA inspected the sprouter X facility on February 12, 1999, and determined that alfalfa sprouts grown during the outbreak period came from a single seed lot (lot L). Trace-forward information from the seed distributor and interviews with the other area sprout growers confirmed that sprouter X was the only grower in the Pacific Northwest to have purchased lot L seeds. Moreover, the illness onset date for the Oregon index case occurred 2 weeks after this sprouter received a shipment of lot L and 1 week after sprouts grown from lot L first reached market.

*S.* Mbandaka was isolated from 11 of 12 alfalfa sprout samples, and one of two lot L seed samples obtained from the sprouter X facility. No pathogens were identified from this sprouter’s samples of radish, onion, and clover sprouts, seed samples from non–lot L seeds, or samples of irrigation water.

Inspectors determined that no seed disinfection had been used at the sprouter X facility. The owner of the facility informed us that disinfection was “unnecessary” because seed samples had tested negative for pathogens before being shipped from the seed broker.

After this inspection, sprouter X issued a voluntary recall of all products. Existing stocks of alfalfa sprouts were impounded, and the company was instructed to cease further production of sprouts using lot L seeds. A press release from the Oregon Department of Human Services was issued on February 12, 1999, advising consumers to return brand X sprouts and retailers to return unsold stocks.

### Genetic Analysis of *S.* Mbandaka Isolates

The PFGE patterns for the *S*. Mbandaka isolates from stool samples from the initial 10 case-patients, the partially consumed box of sprout X retrieved from case-patient 1, and the onsite sprout and seed sample cultures were indistinguishable but clearly differed from the two *S.* Mbandaka reference samples obtained in 1997 and 1998. Of the final 89 cases, 87 had identical PFGE patterns; 2 case-patients included in the total were not tested for technical reasons.

### Trace-Back and Trace-Forward Investigations of Lot L

Sprouter X purchased lot L seeds from a California broker. This broker, in turn, purchased seeds from California seed producer Z. Purchase invoices indicated that 5,454 kg of seed from 18,000 kg of lot L had been distributed among five sprout growers other than sprouter X: sprouters E, F, G, H, and Y (Table). Four sprouters were located in California and one in Florida (sprouter E). Of the 5,454 kg of seed sold to sprouters, 3,864 kg (70.2%) had already been sprouted by the time of the embargo. Of this seed, sprouters X and Y had used 2,610 kg (68%) and sprouters E, F, and G had used 1,254 kg (32%) (Table). Sprouter H had not yet used any lot L seed.

**Table:** Fate of lot L alfalfa seeds

Sprouter (state)	Quantity purchased (kg)	Quantity sprouted (kg)	Seed disinfection	Linked cases	
Sprouter X (Washington)	909	860	None	68	
Sprouter Y (California)	1,818	1,750	None	21	
Sprouter E (Florida)	909	909	20,000 ppm Ca(OCl)_2_	0	
Sprouter F (California)	909	45	20,000 ppm Ca(OCl)_2_	0	
Sprouter G (California)	450	300	500 ppm NaOCl	0	
Sprouter H (California)	450	0	ND^a^	0	
Totals	5,454	3,864			

^a^ND, not determined. The method of disinfection did not apply to sprouter H because this sprouter had not used any lot L seeds by the time of the embargo.

Of the five sprouters who used lot L seeds, two were linked to the *S*. Mbandaka outbreak. Isolates from Washington, Oregon, and Idaho were traced to sprouter X in Washington State; all California cases were traced to sprouter Y. Neither sprouter X nor Y had used seed disinfection during the outbreak period. Of the three sprouters (E, F, and G) using lot L seed but not linked to cases of *S.* Mbandaka, all reported disinfecting their seeds with 2,000–20,000 ppm Ca(OCl)_2_, or 200 ppm sodium hypochlorite (Table).

All seeds from lot L came from a single farm in California’s Imperial Valley. Although we were unable to determine how the seeds became contaminated, an inspection of the farm showed numerous opportunities for contamination by wild and domestic animals or river water.

## Discussion

The epidemiologic, laboratory, and environmental evidence shows that alfalfa sprouts germinated from lot L seeds were the cause of this outbreak. The fact that two geographically distant sprouters (X and Y) were linked to cases of *S*. Mbandaka suggests that the seeds became contaminated before distribution to sprouters. Given the historic underreporting of salmonellosis, the 89 confirmed cases of *S*. Mbandaka probably represent several thousand cases (*24*). Salmonellosis is typically most prevalent in patients <1 year of age (*25*,*26*), so the fact that women 19–41 years of age predominated in this outbreak is noteworthy. This pattern was observed in several previous sprout-associated outbreaks (*3*,*27*–*30*). We believe this pattern reflects the demographics of sprout consumers and does not imply that women of this age range are inherently predisposed to salmonellosis in general or to *S*. Mbandaka in particular. As noted in previous outbreaks, the urinary tract was a common site of infection among women (*28*).

The outbreak was limited to consumers of sprouts grown by two sprouters who used the same seed lot. Neither sprouter used the FDA-recommended seed-disinfection process. Of the three sprouters who disinfected their seeds, none were linked to cases, although they had used 32% of the lot L seed that was sprouted. This finding suggests that seed disinfection does reduce the risk for human disease posed by seed sprouts.

Since a formal randomized trial of seed disinfection in the community would be unethical, our understanding of the usefulness of disinfection is based largely on observations made in the course of outbreak investigations. In the current study, this outbreak fortuitously served as a natural experiment of the effectiveness of seed disinfection.

Few other published outbreak investigations have addressed the value of disinfection at preventing human disease. Clover sprouts were implicated in a 1999 outbreak in Colorado (*31*). The two sprouters involved also grew sprouts from a common seed lot, though only one sprouter reported followed the FDA’s disinfection guidelines. Salmonellosis linked to the sprouter using disinfection occurred at a rate of 0.29 cases per 50 kg bag of seed versus 1.13 cases per 50 kg bag from the sprouter not using disinfection. The investigators concluded that disinfection reduced, but did not eliminate, the risk for human salmonellosis.

In another recent outbreak, Proctor et al. investigated a multistate alfalfa sprout–associated salmonellosis outbreak in which 20,000 ppm Ca(OCl)_2_ disinfection was used (*30*). This outbreak was confined to a single sprouter; in the absence of a “control” sprouter not using disinfection, ascertaining whether disease rates would have been even higher is impossible. Nevertheless, both outbreaks amply demonstrate that disinfection is an imperfect remedy for the problem of contaminated seed sprouts.

The inoculum size needed to cause infection is known to vary between different serotypes of *Salmonella* (*32*–*34*). Because disinfection reduces but does not eliminate pathogens, this variation might account for some of the apparent disinfection failures if levels were not reduced below some critical threshold. Human error may also be to blame. In a recent survey of disinfection practices of area mung-bean and green sprouters (green sprouts include alfalfa, clover, radish, and onion sprouts) who reported observing the FDA guidelines, California investigators noted that six of seven green sprouters and one of nine mung-bean sprouters succeeded in reaching the target of 20,000 ppm Ca(OCl)_2_ (J. Thomas, unpub. data). Accordingly, future investigations should include careful reviews of the disinfection records of implicated sprouters. Receipts for seed-disinfection chemicals, protocols for disinfection, and disinfection logs may prove useful clues to actual disinfection practices. Asking the sprouter to simulate the disinfection process may also be instructive.

Given these limitations, a more reliable method of decontamination is needed if eating sprouts is to be safe. Gamma irradiation of sprout seeds or mature product reduces or eliminates colonizing bacteria. However, the prohibitive expense of irradiating machinery places this technology beyond the means of most sprouters. Moreover, irradiating seeds reduces the germination yield and affects the appearance of mature sprouts that germinate (*35*,*36*). Marketing irradiated sprouts to health-conscious consumers may prove challenging, given the frequent misconception that irradiated sprouts are themselves radioactive. A concerted public awareness campaign might avoid consumer anxiety about this process. Sprouters could also pool resources towards the collective purchase or use of irradiating machines. Cooking sprouts is highly effective at eliminating pathogens but is not a popular remedy because sprouts are typically consumed raw.

The pathogen detection assay used at the request of sprouter X did not detect the *S.* Mbandaka contaminating the lot L seeds. The test used a proprietary protocol wherein several grams of seed were randomly sampled for testing from each 50-kg bag of seed by using a monoclonal antibody-based enzyme-linked immunoassay (Silliker Laboratories, pers. comm.). Regardless of the assay’s sensitivity, this technique appears highly vulnerable to sampling error unless the pathogen is uniformly distributed throughout the seed. Notably, research to date indicates that pathogens are dispersed heterogeneously and at low densities on seeds (*37*,*38*). Compounding this vulnerability, the assay used was designed only to detect *Salmonella*, a shortcoming given that enterohemorrhagic *E. coli* outbreaks linked to sprouts have occurred repeatedly (*28*,*39*–*43*).

In the current outbreak, a negative test was cited by the sprouter X’s proprietor as the rationale for disregarding FDA recommendations. This failure is noteworthy given that *S.* Mbandaka was readily isolated from seeds both by the Oregon State Health laboratory and an independent lab in California (*44*), by using more sensitive enrichment and detection processes. Currently, FDA does not advocate routine seed screening, although it does recommend testing sprouts or spent irrigation water samples for enteric pathogens (*18*), neither of which was performed by sprouter X.

For the time being, FDA should continue to promote adherence with its guidelines for disinfecting seeds and testing sprouts and irrigation water. The International Sprout Grower’s Association (ISGA), a private trade group, has responsibly encouraged adherence among its members by allowing sprouters who use 20,000-ppm Ca(ClO)_2_ disinfection and pass a third-party production inspection to label their sprouts as ISGA-certified. However, this precaution has yet to be translated into a marketing advantage through retailer and consumer education; compliance remains voluntary, and no systematic data exist indicating the proportion of sprouters who adhere to the guidelines. Adherence with a uniform disinfection process might be improved if made subject to regulation or if retailers were to insist on “ISGA-certified treated seed” in their purchase contracts for sprouts.

Given repeated outbreaks, seed testing that is falsely reassuring, ineffectiveness and incompleteness of seed disinfection, and a lack of commitment to disinfected product from retailers, we think that consistently rendering raw sprouts free of enteric pathogens is not practical. Persons at increased risk for invasive salmonellosis and those wishing to reduce their risk for foodborne infections may be well advised to avoid sprouts entirely.
